# Extension of the SUGRES-1P Coarse-Grained Model of Polysaccharides to Heparin

**DOI:** 10.1021/acs.jctc.3c00511

**Published:** 2023-08-16

**Authors:** Annemarie Danielsson, Sergey A. Samsonov, Adam Liwo, Adam K. Sieradzan

**Affiliations:** Faculty of Chemistry, University of Gdansk, ul. Wita Stwosza 63, 80-308 Gdańsk, Poland

## Abstract

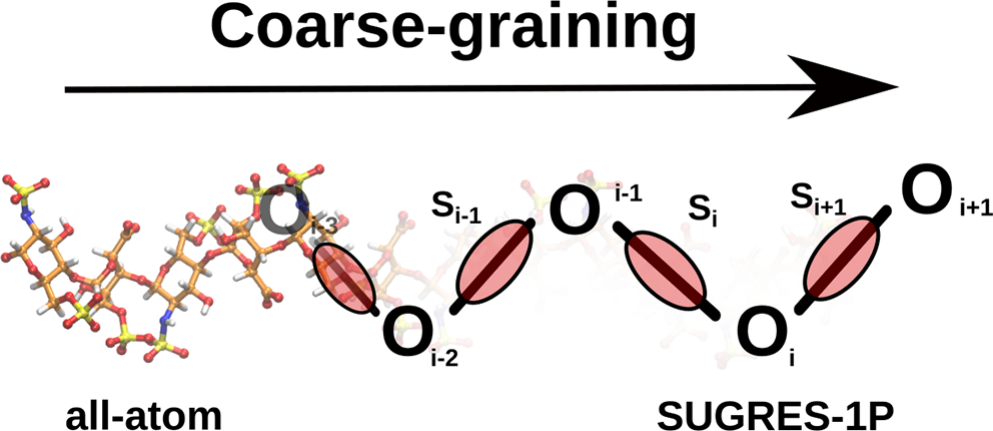

Heparin is an unbranched periodic polysaccharide composed of negatively charged monomers and involved in key biological processes, including anticoagulation, angiogenesis, and inflammation. Its structure and dynamics have been studied extensively using experimental as well as theoretical approaches. The conventional approach of computational chemistry applied to the analysis of biomolecules is all-atom molecular dynamics, which captures the interactions of individual atoms by solving Newton’s equation of motion. An alternative is molecular dynamics simulations using coarse-grained models of biomacromolecules, which offer a reduction of the representation and consequently enable us to extend the time and size scale of simulations by orders of magnitude. In this work, we extend the UNIfied COarse-gRaiNed (UNICORN) model of biological macromolecules developed in our laboratory to heparin. We carried out extensive tests to estimate the optimal weights of energy terms of the effective energy function as well as the optimal Debye–Hückel screening factor for electrostatic interactions. We applied the model to study unbound heparin molecules of polymerization degree ranging from 6 to 68 residues. We compare the obtained coarse-grained heparin conformations with models obtained from X-ray diffraction studies of heparin. The SUGRES-1P force field was able to accurately predict the general shape and global characteristics of heparin molecules.

## 1 Introduction

Glycosaminoglycan (GAG) heparin (HP) is a linear, anionic polysaccharide composed of repeating disaccharide units of 2-*O*-sulfated iduronic acid (IdoA2S) and 6-*O*-sulfated and *N*-sulfated glucosamine (GlcNS6S).^1^ With a −4 net charge per disaccharide unit, HP is the most negatively charged polysaccharide among all GAGs. Synthesized in mast cells of connective tissues as part of proteoglycans, after its synthesis HP is stored in secretory granules of mast cells^2^ and released into the extracellular matrix, where it is involved in a range of important biological processes, such as angiogenesis,^3,4^ anticoagulation,^5^ cell proliferation,^6^ cell adhesion,^7,8^ and cell migration.^9−11^ HP fulfills its role via interactions with protein partners. The disruption of protein–HP interactions can lead to the development of diseases and pathologies, including tumor growth and metastasis,^3,12−14^ neurodegenerative,^15−18^ prion diseases,^19−21^ and autoimmune disorders.^22,23^ The participation of HP in essential biological processes and pathways renders it a promising and interesting target in medicine.^24^

The structure and dynamics of HP as well as its interactions with proteins have been investigated by various experimental techniques, including nuclear magnetic resonance (NMR),^25−28^ X-ray diffraction,^29−32^ surface plasmon resonance,^33−35^ mass spectrometry,^36,37^ and capillary electrophoresis.^38−41^ However, experimental techniques face a range of challenges in the study of HP and other GAGs, mainly stemming from the periodicity,^42^ considerable length, and high molecular weight of native GAGs,^43−45^ diversity of sequences and sulfation patterns,^44^ conformational flexibility,^46^ and tendency to cause oligomerization and precipitation of proteins.^45^

To fully elucidate the structure and dynamics of biomolecules, computational approaches are often used alongside experimental techniques.^47,48^ A standard computational method is all-atom molecular dynamics (MD) simulations, in which Newtonian laws of motion are applied to determine the movement of each individual atom of the simulated system over time.^47,48^ The trajectories resulting from MD simulations, therefore, show the dynamic behavior of biomolecules and their complexes. MD simulations of HP molecules of different lengths were used to study their conformation in solution^49,50^ and in complexes with proteins.^51−54^

While MD simulations are invaluable in the elucidation of the three-dimensional structures of GAGs and the atomistically detailed mechanisms of binding with proteins, they might also fail to produce satisfactory results. Native GAG chains can achieve sizes of up to 100 kDa (degree of polymerization up to approximately 30,000,000)^55^ and are, therefore, too large to be simulated using classical all-atom MD approaches. The addition of protein receptors, solvents, and ions increases the system size even when modeling only short HP fragments, which reduces the timescales that can be covered by all-atom MD techniques. The length of HP molecules together with their high flexibility result in a large conformational space that may be sampled insufficiently. Additionally, the binding of GAGs by proteins is predominantly electrostatic in nature and GAG-binding sites are most often patches of positively charged amino acid residues on the protein surface, as opposed to deep binding pockets.^56,57^ This, as well as multipose binding, a phenomenon in which multiple binding poses of comparable binding energy coexist, causes difficulties in the determination and prediction of the exact binding poses of GAGs in protein-GAG systems.^58,59^

An alternative to classical all-atom MD methods that allows the coverage of larger spatial and temporal scales is coarse-grained (CG) modeling. CG approaches are based on a reduction of the representation of a system studied by introducing the so-called pseudoatoms that represent groups of atoms. When compared to all-atom MD representations, coarse graining of the system representation significantly reduces the number of degrees of freedom. As a result, the computational resources required to simulate the system are reduced. At the same time, the reduction in resolution is inherently accompanied by a reduction in detail and accuracy, so care has to be taken in the construction of the CG model in order to keep key features of the system and retain the essential characteristics of the biomolecules.^60^

In spite of the significance and ubiquity of GAGs in the biological context, only a handful of CG models have been developed for this class of molecules, possibly due to the complexity of their sequence and structure.^61^ The first CG model of GAGs was proposed by Bathe et al. in 2005,^62^ designed for the modeling of chondroitin, chondroitin sulfate, and hyaluronic acid. The sugar residue was represented by a total of five CG beads: two carbon atoms and an oxygen atom to model the glycosidic linkage, an interaction site corresponding to the center of mass, used to model steric interactions, and an interaction site corresponding to the center of charge, modeling the electrostatic interactions. The model was able to correctly reproduce the conformation of the GAGs studied as well as their titration characteristics.^62^

Sattelle et al.^63,64^ designed a CG model of heparan sulfate by reducing the representation of the sugar residue to two interaction centers: one representing the sugar ring and the second one representing the glycosidic linkage oxygen atom. The model was successfully applied to the study of heparan sulfate of large size^64^ and heparan sulfate-containing proteoglycans.^63^

A more detailed model, comprising 28 different pseudoatoms for the simulation of 17 different GAG types was proposed by Samsonov et al.^65^ The pseudoatoms corresponded to different functional groups of the GAGs, including oxygens of the glycosidic linkages, sulfate groups, the carboxylate and *N*-acetyl groups, centers of mass of the pyranose rings and the CH_2_OH group of the C_6_ atom of *N*-acetyl-glucosamine and *N*-acetyl-galactosamine. The number of CG pseudoatoms per repeating unit ranged from 2 to 5 depending on the type and sulfation pattern of the sugar residue (*N*-acetyl-galactosamine/*N*-acetyl-glucosamine and glucuronic acid/iduronic acid) and its location in the GAG chain (internal/terminal). Geometrical parameters for virtual bonds, virtual bond angles, and virtual bond torsional angles were obtained from all-atom MD simulations of different GAGs, while parameters of nonbonded interactions were obtained using steered MD. Pseudoatoms representing carboxyl and sulfate groups were assigned charges equal to −1, while all other pseudoatom charges were set to 0. The CG model achieved good results in the modeling of global and local characteristics of GAG chains of different lengths.^65^

Another CG model of GAGs is based on the SUGRES-1P model of carbohydrates, which is a part of the UNIfied COarse gRaiNed (UNICORN) model for biomacromolecules.^66,67^ The UNICORN model relies on a strictly physics-based approach, reducing the representation to only one or two CG sites per repeating unit depending on the type of macromolecule, and the effective energy function of the modeled system.^68^ The transferability of the model to different systems is ensured by the decomposition of the potential of mean force of a system into a sum of contributions from its parts, corresponding to interactions within the CG sites, pairs of CG sites, as well as groups of CG sites. The UNICORN model has been successful in the prediction of protein structures,^69^ folding kinetics,^70^ conformational changes,^71^ and RNA and DNA structure and dynamics.^72^

In this study, we present the implementation and calibration of the SUGRES-1P CG model.^67,73^ We based our implementation on the theoretical background and initial parametrization of the sugar rings with each other published by our group.^67,73^ Furthermore, we have fine tune the weights and parameters of the force field and applied the SUGRES-1P force field to the simulation of free HP of degree of polymerization (dp) ranging from 6 to 68. The conformations obtained from CG simulations were compared to experimentally determined HP structures.^74,75^ The results show a good agreement with the experimental data in terms of the general shape and conformation of the CG HP molecules as well as their global characteristics: end-to-end distance (EED) and radius of gyration (*R*_g_).

## 2 Methodology

### 2.1 SUGRES-1P CG Force Field

**Figure 1:**
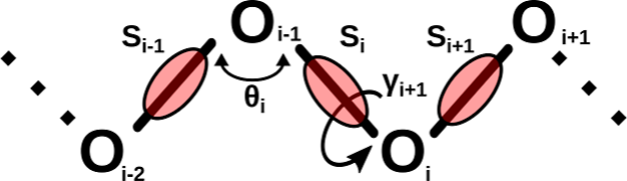
Illustration of the SUGRES-1P model. The interaction sites are united sugar rings, represented by transparent red ellipsoids, located half-way between glycosidic oxygen atoms (white spheres) which are not interaction sites but serve to define the geometry of the polysaccharide molecule. The virtual bonds connecting the oxygen atoms are shown as thick black lines. The geometry of the polysaccharide chain is defined by the virtual bond angles θ_*i*_ and torsional angles γ_*i*_.

The SUGRES-1P model^67,73^ employed in this work is a physics-based model of polysaccharide chains which is a part of the CG UNICORN model of biomolecules, alongside the UNited RESidue (UNRES), and united Nucleic Acid RESidue (NARES-2P) models for polypeptides and nucleic acids, respectively.^68^ In the SUGRES-1P model, the polysaccharide chain representation is reduced to a single interaction site per sugar residue located halfway between glycosidic linkage oxygen atoms, which serve as anchor points of the polysaccharide chain (Figure 1). The geometry of the polysaccharide chain in the SUGRES-1P model is defined by virtual bonds (corresponding to O1 → O4 glycosidic linkages), the virtual bond angles θ_*i*_, and virtual bond dihedral angles γ_*i*_, as shown in Figure 1.

Each interaction center of the polysaccharide consists of two sections, termed the “head” and “tail”. This is brought by the necessity of accounting for the anisotropy of the CG HP residues, as the center of the charge is off the geometrical center of the residue. Therefore, the location of the head corresponds to the center of the charge, while that of the tail corresponds to the uncharged part of the residue. As a result, the energy of interactions between HP residues is calculated as a sum of the head–head, tail–tail, and head–tail interaction energies. The SUGRES-1P physics-based effective energy function of a polysaccharide chain is expressed by eq 1
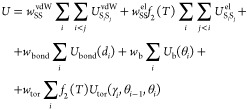
1

The term *U*_bond_(*d*_*i*_) accounts for the energetics of the virtual-bond-deformation, where *d*_*i*_ corresponds to the length of the *i*-th virtual bond, *U*_b_(θ_*i*_) is the virtual bond angle deformation term for angle θ_*i*_, and *U*_tor_(γ_*i*_, θ_*i*–1_, θ_*i*_) is the virtual-bond-torsional energy term for virtual bond dihedral angle γ_*i*_, where angles (θ_*i*–1_ and θ_*i*_) are the adjacent virtual bond angles.

The term  is represented by a sum of energy terms of the interaction of the polar and charged parts of the residue, excluding the Coulombic charge–charge interactions, as expressed by eq 2
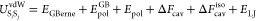
2

The interaction between the uncharged parts of the interaction sites is modeled using the Gay–Berne potential,^76^ expressed by
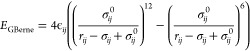
3where *r*_*ij*_ is the distance between the uncharged tails of the interacting particles, σ_*ij*_ is the distance corresponding to the zero value of *E*_GBerne_ for arbitrary orientation of the particles (σ_*ij*_^0^ is the distance corresponding to the zero value of *E*_GBerne_ for the side-to-side approach of the particles), and ϵ_*ij*_ is the van der Waals well depth.

The contribution to the energy arising from polarization of the solvent, *E*_pol_^GB^, by charged parts of the interaction sites is computed using the generalized Born model
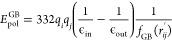
4where *q*_*i*_, *q*_*j*_ are the charges of the interacting particles, ϵ_in_ is the effective dielectric constant of the “inside” of the interacting particles, ϵ_out_ is the effective dielectric constant of the solvent, *r*_*ij*_′ is the distance between the charged heads of the interacting particles, and *f*_GB_(*R*) is the Generalized Born function expressed by
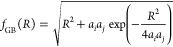
5where *a*_*i*_ and *a*_*j*_ are Born radii and *R* corresponds to the distance between the given sites of the interacting particles (e.g., the distance between the charged heads, *r*_*ij*_′, in the case of *E*_pol_^GB^).

*E*_pol_ is the polarization energy corresponding to the interactions between the charged and uncharged parts of the interaction sites of two sugar residues
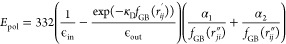
6where *r*_*ij*_′ is defined under eq 3, *r*_*ji*_″ is the distance between the uncharged tail of particle *i* and the charged head of particle *j*; *r*_*ij*_″ is the distance between the head of particle *i* and the tail of particle *j*; ϵ_in_ and ϵ_out_ are dielectric constants within the particles and in bulk, respectively; κ_D_ is the length of Debye screening due to the presence of counterions; and α_1_ and α_2_ are solvation parameters of the tails of the particles.

The cavity term of the isotropic charged heads is expressed by Δ*F*_cav_^iso^
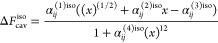
7with
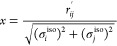
8

In the above equations, *r*_*ij*_′ is the distance between two charged parts of the interaction site of particles *i* and *j*, and σ_*i*_^iso^ and σ_*j*_^iso^ are equivalent to the minimum distance between the center of charge of particle *i* or *j*, respectively.

The cavity term of the uncharged tails is calculated by
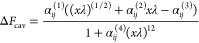
9where
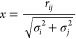
10and
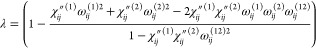
11where *χ*_*ij*_″^(1)^ and *χ*_*ij*_″^(2)^ are anisotropies related to Δ*F*_cav_, *r*_*ij*_ is the distance between the tails of the interacting particles, and σ_*i*_ and σ_*j*_ are calculated with the minimum distance between the centers of the interacting particles.

The isotropic Lennard-Jones potential is used to model the van der Waals interaction energy between two polar heads
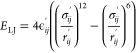
12where *r*_*ij*_′ is the distance between the polar heads, σ_*ij*_′ is the distance corresponding to the zero value of *E*_LJ_, and ϵ_*ij*_′ is the van der Waals well depth.

The electrostatic interaction energy between the charged parts of the interaction sites is expressed by
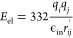
13

The energy terms are multiplied by the weights *w* as well as, in the case of , temperature factors *f*_*n*_(*T*) that reflect the temperature dependence of the appropriate effective-energy terms, defined by
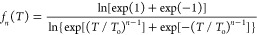
14where *T*_o_ = 300 K.

For a more detailed description of the CG SUGRES-1P force field, the reader is referred to refs (67) and (73).

### 2.2 HP Structures

The NMR structure of HP from PDB: 1HPN(25) was used as a template for the construction of HP fragments used in CG simulations. HP molecules of length from 6 to 68 residues, which are referred to as dp6, dp8, dp10, dp12, dp14, dp16, dp18, dp24, dp30, dp32, dp36, dp48, and dp68, respectively, were constructed using the xLeap module of AMBER^77^ by either elongating or shortening the template structure. The choice of HP molecule lengths was dictated by the availability of experimental data used as a reference to compare with the results obtained using the CG MD simulation.^74^

As a reference, measures of EED and radius of gyration (*R*_g_) from analytical centrifugation and synchrotron X-ray scattering experiments for HP dp6, dp12, dp18, dp24, dp30, and dp36^74^ and HP dp32, dp48, and dp68^75^ were used. The conformations of HP oligomers obtained from CG simulations were compared with the structures of HP dp18, dp24, dp30, and dp36 obtained by^74^ using constrained scattering modeling based on the structure of HP in ref (25).

### 2.3 Estimation of Energy-Term Weights and the Debye–Hückel Screening Factor

Due to the high negative charge of HP,^1^ the inclusion of an appropriate amount of counterions in simulations is necessary. In the SUGRES-1P model, this is possible by the adjustment of the Debye–Hückel screening factor κ^–1^, which describes the electrostatic screening distance of charges in an electrolyte^78,79^ and is linked to the ionic strength of a solution, as shown in eq 15, for the case of monovalent ions
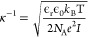
15where κ^–1^ is the Debye–Hückel screening length, ϵ_r_ is the dielectric constant of water, ϵ_0_ is the vacuum dielectric permittivity, *k*_B_ is the Boltzmann constant, *T* is the absolute temperature, *N*_A_ is the Avogadro number, *e* is the elementary charge, and *I* is the ionic strength of the electrolyte.^79^ The Debye–Hückel length κ^–1^ is part of the  term in eq 6 in the SUGRES effective energy equation. The expression for  has been adapted from previous studies of the effective interactions of like-charged side chains in the UNRES force field.^80^ While a direct comparison between values of κ^–1^ as used in SUGRES-1P and actual ionic strength of solutions cannot be made due to the implicit nature of the solvent in the UNICORN CG model, the value of κ can be expected to increase with the square of ionic strength, as can be deduced from eq 15.

The appropriate weights of the energy terms from eq 1, as well as the optimal value of the Debye–Hückel screening factor, were determined empirically using HP of three different lengths (HP dp12, HP dp24, and HP dp68). For each effective-energy term, the corresponding weight was increased from 1 to 10 with increments of 1. At the same time, for each examined set of energy term weights, the value of the κ parameter was increased from 0.0 to 1.0 with increments of 0.1, as detailed in Table S1, emulating the increase of salt concentration.

Canonical Langevin dynamics simulations of each HP molecule were conducted with the SUGRES-1P force field for all combinations of effective energy-term weights and κ values without any restraints imposed on the size of the molecule, starting from the extended conformation. The time step size stems from the energy and distance units conversion from kcal/mol and Å, respectively (molecular time unit). Previous publications have investigated the influence of the step length used in the MD algorithm of the UNRES force field,^81,82^ especially in the context of energy drift in the CG simulations. The time step equal to 4.89 fs was recommended as a safe value for the stability of the MD algorithm.^82^ To obtain approximately 1 μs real time per simulation, the simulations in our work were therefore carried out for 2,000,000 steps per simulation. The RMSD of the HP molecules in reference to the starting structures has been inspected to ensure the convergence of the MD simulations. The EED and radii of gyration (*R*_g_) of the obtained HP conformations were compared to experimental data.^74,75^

All CG simulations have been conducted at *T* = 300 K. Due to the dependence of the energy function employed by the SUGRES-1P force field on the temperature, a change of the simulation temperature would likely affect the obtained results and the choice of optimal parameters. Nevertheless, all simulations were conducted at *T* = 300 K as it represents a temperature close to the physiological temperature and corresponds to the experimental conditions employed most commonly in experiments, including the ones we used as a reference for our data.^74^

### 2.4 CG Simulations of Short and Long Free HP Molecules for Selected Weights of Effective Energy Terms and Selected Values of κ

Canonical Langevin CG dynamics were conducted for HP dp6 to dp68 using three combinations of κ and weights of the effective energy terms:

1. κ_2_ and electrostatic interaction energy weight (*w*_eel_) equal 7,
2. κ_7_ and electrostatic interaction energy weight (*w*_eel_) equal 7,
3. κ_7_ and virtual bond-stretching energy weight (*w*_bond_) equal 4.

The trajectories comprised 2,000,000 steps with a 4.89 fs step length. The EED and radii of gyration (*R*_g_) of the obtained HP conformations were compared to experimental data.^74,75^ The overall conformations of CG HP dp18, dp24, dp30, and dp38 were compared to the conformation obtained by ref (74) using constrained scattering modeling based on the NMR structure of HP.^25^

### 2.5 Visualization and Analysis

The CG conformations of HP were visualized using VMD^83^ and analyzed using the cpptraj module of AMBER.^77^ Comparisons of EED and *R*_g_ values as well as their visualization were performed using R.^84^ Clustering of frames of the CG trajectory has been performed using the DBSCAN^85^ algorithm using the cpptraj module of AMBER^77^ with a minimum cluster size of 2 and distance cutoff of 4 Å.

## 3 Results and Discussion

### 3.1 Estimation of Energy Term Weights and the Debye–Hückel Screening Factor

An initial CG simulation of free HP using default effective-energy term weights and parameters determined by refs (67) and (73) did not yield satisfactory results. The HP molecules formed tight coils, which are not likely to correspond to in vivo conformations.

**Figure 2:**
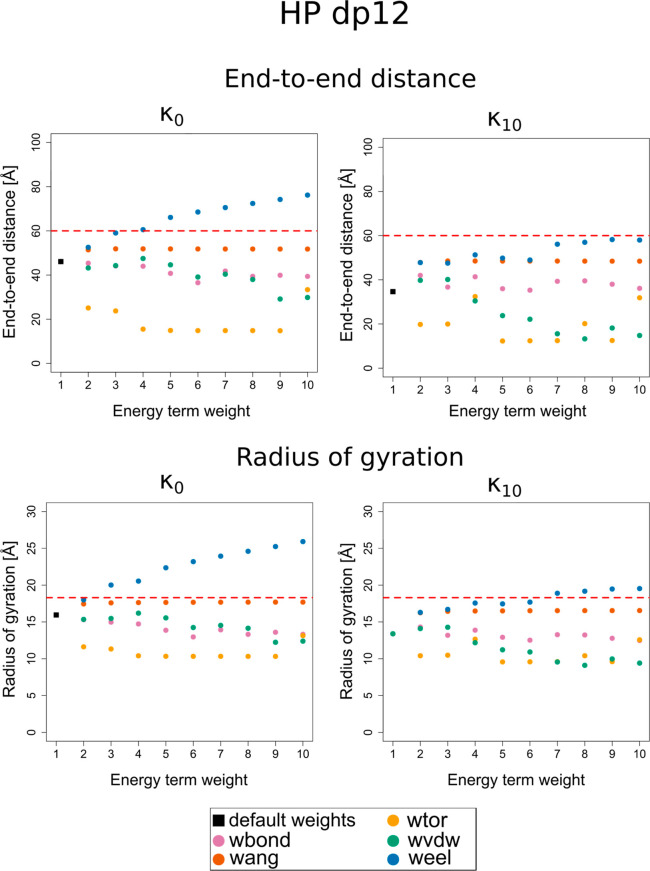
Dependence of the EED and the radius of gyration (*R*_g_) on the energy term weight and ion concentration expressed by the κ parameter for HP dp12. The black square corresponds to all weights set to 1 (default) while colored circles (color legends shown below the graph) correspond to varying a given weight from 1 to 10 with a step of 1. See eq 1 and the text below this equation for the energy-term and energy-term-weight symbols. The experimental values of EED and *R*_g_ from refs (74) and (75) are shown as dashed red lines.

**Figure 3:**
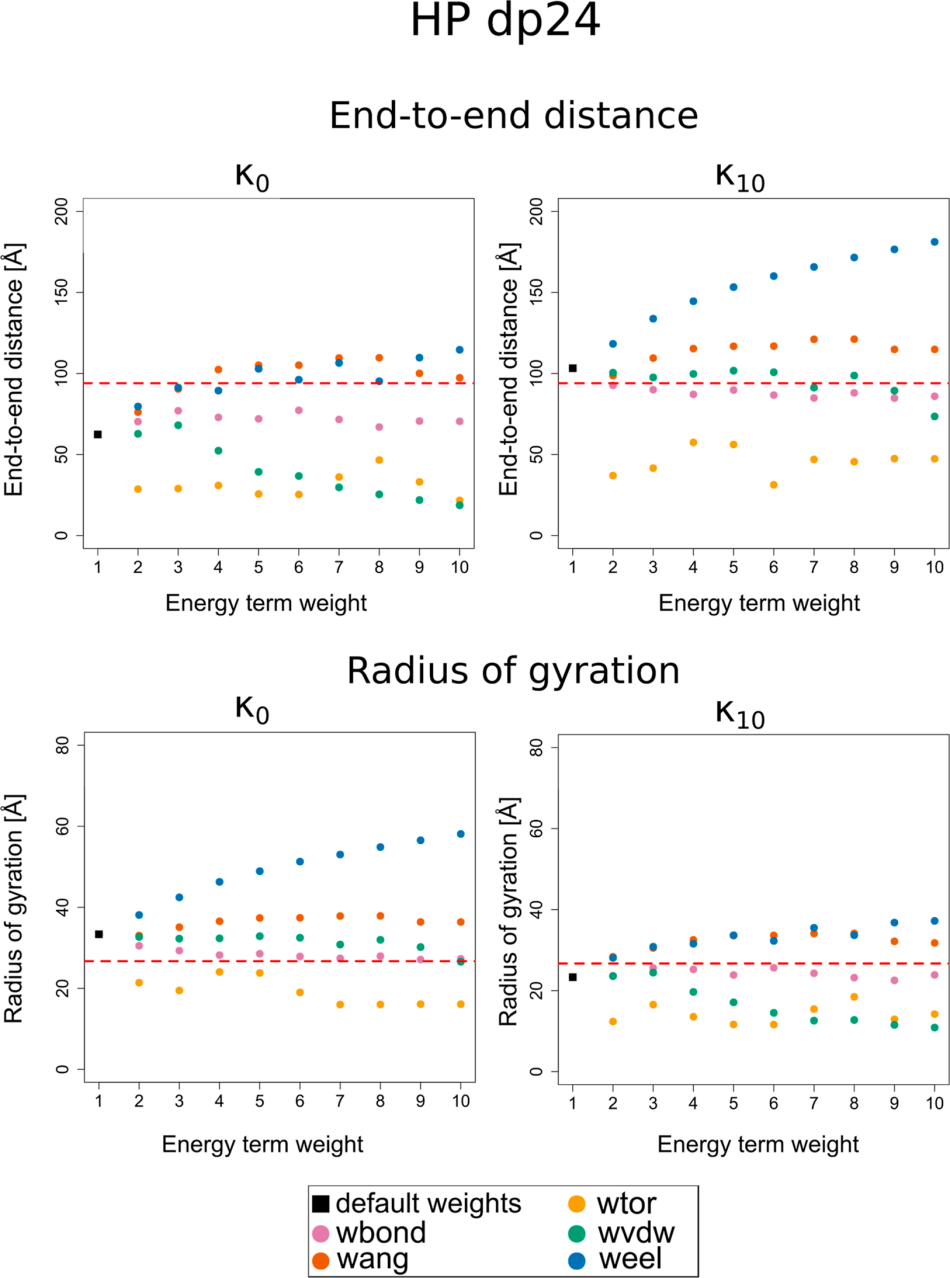
Dependence of the EED and the radius of gyration (*R*_g_) on the energy term weight and ion concentration expressed by the κ parameter for HP dp24. The black square corresponds to all weights set to 1 (default) while colored circles (color legends shown below the graph) correspond to varying a given weight from 1 to 10 with a step of 1. See eq 1 and the text below this equation for the energy-term and energy-term-weight symbols. The experimental values of EED and *R*_g_ from refs (74) and (75) are shown as dashed red lines.

**Figure 4:**
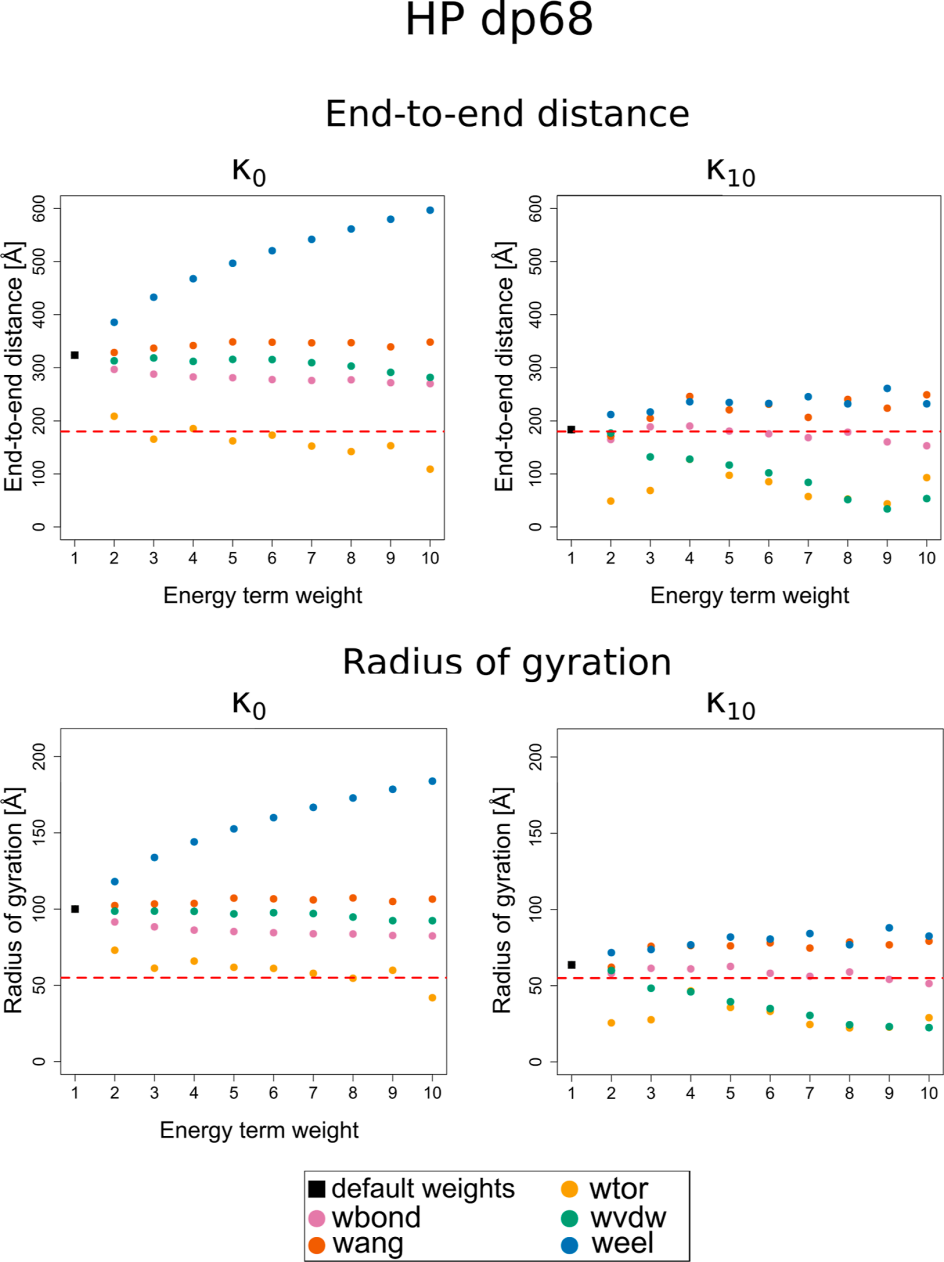
Dependence of the EED and the radius of gyration (*R*_g_) on the energy term weight and ion concentration expressed by the κ parameter for HP dp68. The black square corresponds to all weights set to 1 (default) while colored circles (color legends shown below the graph) correspond to varying a given weight from 1 to 10 with a step of 1. See eq 1 and the text below this equation for the energy-term and energy-term-weight symbols. The experimental values of EED and *R*_g_ from refs (74) and (75) are shown as dashed red lines.

The optimal weights of the effective energy terms from eq 1 as well as the optimal value of the κ parameter accounting for counterion screening have been determined empirically by conducting CG simulations of HP dp12, dp24, and dp68 for all combinations of energy term weights and κ as described in the Methodology section. In short, each energy-term weight was increased from 1 to 10 with a step of 1 while keeping all other energy-term weights at a default value of 1. For every considered value of the weights, the value of κ was increased from κ_0_, corresponding to a total lack of counterions in the simulated system, to κ_10_, as detailed in Table S1. The average EED and *R*_g_ values across the CG simulation trajectory were compared to experimental values from refs (74) and (75) in order to determine which combination of weights and κ resulted in conformations with characteristics most similar to the experimental HP conformations. Figures 2–4 summarize the findings for HP dp12, dp24, and dp68 for κ_0_ and κ_10_, which correspond to the two extremes of the tested values, i.e., a complete lack of ions and high salinity of the simulated system.

The increase of the κ parameter, corresponding to an increase in the ion concentration of the implicit solvent, led to a decrease in the EED and *R*_g_ of the three examined HP molecules. The same effect, i.e., the adoption of more coiled conformations, was achieved by increasing the weight of the torsional energy term. On the other hand, more elongated molecules were obtained by increasing the weight of the electrostatic energy term. Such conformations were also characterized by a larger radius of gyration.

Depending on the chain length of the analyzed HP molecules, the structures obtained from CG simulations were in good agreement with experimental data at different ion concentrations.^74,75^ Interestingly, the experimental EED of the short HP dp12 lied within the range of achievable values for simulations conducted in the absence of ions (i.e., for κ_0_). The percentage of error (PE) of EED ranged from 75.3% below to 73.0% above the experimental value of 60 Å. In contrast, the EED of CG HP dp12 simulated in κ_10_ achieved values close to the experimental EED only in the case of very high weights of the electrostatic energy term, with the closest value lying 2.9% below the experimental EED. While less pronounced than in the case of EED, the same can also be observed for the *R*_g_ of HP dp12. The experimental EED and *R*_g_ of HP dp24 were within the ranges of EED and *R*_g_ values of the simulated HP molecules for all the considered κ_*i*_ values. In general, the obtained HP dp68 conformations were characterized by an EED and *R*_g_ of the chain closer to the experimentally determined EED and *R*_g_ in higher ion concentrations compared to the shorter HP molecules. The long HP dp68 chains have a higher propensity for forming coiled conformations owing to their length; hence, the EED of the simulated chain is probably controlled less by the repulsive electrostatic forces and more by the increased flexibility of the virtual bonds and virtual bond angles caused by an increased weight of the bond stretching and torsional angle deformation energies.

The need for the adjustment of the energy-term weights is evident, as the use of default weights resulted in either too compact or too extended molecules compared to the experimentally determined conformations. At the same time, the different preferences for ion concentration and energy term weights for short and long HP chains highlight the challenge of finding one universal set of parameters and weights for all lengths of HP. The choice of optimal energy-term weights and κ_*i*_ employed in this study relied on an empirical approach that resulted in a set of parameters enabling the simulated HP molecules to adopt conformations similar to the ones observed in experiments (e.g., in ref (74)). While this approach proved successful in this study, due to the interdependence of the parameters of the force field, more advanced optimization techniques may be applied in future developments of the SUGRES-1P force field. Recent research showed the successful application of a range of sophisticated optimization approaches, including a variety of machine-learning algorithms.^86−94^ While such complex parametrization techniques are without a doubt an interesting approach in possible future developments of the SUGRES-1P force field, for the aim of proof of concept of this study, we have restricted ourselves to the empirical approach. For this purpose, we have determined 10 combinations of energy term weights and κ_*i*_ for each of the three tested HP lengths that resulted in the best agreement with experimental EED and *R*_g_ values from refs (74) and (75) (detailed in Tables S2–S4).

Simulations at lower ion concentrations (κ_*i*_ ranging from κ_0_ to κ_5_) together with an increased weight of the electrostatic energy term (*w*_eel_ from 3 to 10) resulted in only small discrepancies between the experimental EED and the EED of CG HP dp12 molecules (Table S2). The percentage error of the EED of CG molecules ranged from 1.6% below to 1.9% above the experimental value, with the closest EED lying just 0.2% above the experimental EED in the case of κ_2_ and *w*_eel_ equal 7. The *R*_g_ of the conformation obtained in simulations with these settings were, however, higher than the corresponding experimental *R*_g_ values (discrepancies within 12.6% of the experimental EED value). In order to reduce the difference between the CG *R*_g_ and the experimental *R*_g_, simulations had to be conducted at low ion concentrations (κ_0_ to κ_6_) together with the electrostatic energy term weights between *w*_eel_ = 2 and *w*_eel_ = 6. While reducing the percentage error of *R*_g_ values to a maximum of 2.2%, this increased the discrepancies in the EED values (a percentage error of the values of up to 17.3%). Consequently, the HP dp12 molecules simulated in settings better fitted to replicate experimental *R*_g_ values were shorter than the HP molecules studied by ref (74).

The 10 HP dp24 conformations with EED values closest to the experimental lengths were obtained from CG simulations in a larger range of κ_*i*_ values than in the case of HP dp12 (κ_*i*_ ranging from κ_0_ to κ_10_). The simulated molecules were characterized by EED lying within 1.5% of the experimentally determined value. When the EED values of the simulated molecules were closer to the experimental EED values, the *R*_g_ values were below the experimentally determined *R*_g_ values (percentage error up to 26.1% of the experimental value). In order to achieve *R*_g_ values closer to those determined by ref (74), the modification of either the virtual bond-stretching energy term weight (*w*_bond_ from 2 to 4), the weight of the van der Waals interaction energy (*w*_vdw_ equal either 4 or 10), or the use of default energy term weights in a low ion concentration (κ_2_) was necessary. As a consequence, however, the HP molecules were shorter than the experimentally- determined conformations (up to 21.8% shorter than the experimental EED). It is important to note that while the EED and *R*_g_ are closely related measures; they describe different aspects of the size of polymer chains. For instance, a coiled polymer chain may be characterized by a small EED, owing to the close location of its ends, but a larger *R*_g_ value due to the overall volume of the coiled chain. Consequently, an inconsistency in the increase rate of the EED and *R*_g_ values of the simulated molecules, depending on the changes of the effective-energy weights and κ_*i*_, could be observed.

Although a wide range of κ_*i*_ values enabled HP dp68 to achieve EED close to the experimental values, a predominance of higher κ_*i*_ among the 10 best combinations of κ_*i*_ and energy term weight could be observed. The conformations obtained from the CG simulations lied within the close range of the experimental EED (the percentage error of the EED lied at and below 0.8% for the 10 best combinations of energy weights and κ). The modification of the virtual bond stretching energy term weight was the most prevalent modification among those resulting in EED values closer to the experimental EED. The HP dp68 conformation characterized by a *R*_g_ closest to the experimental *R*_g_, as determined by ref (75), was obtained by conducting CG simulations without the presence of ions in the implicit solvent together with a modification of the torsional energy term weight to *w*_tor_ = 8 (percentage error 0.6%). This resulted in a more compact and coiled chain, yet at the same time flexible in the context of the rotation of the virtual bonds. The remaining combinations of parameters among those resulting in a better agreement of experimental and theoretical *R*_g_ (percentage error within 1.6%) comprised modifications of the virtual bond stretching energy weights (*w*_bond_ from 6 to 10) in a wide range of κ values (κ_0_ to κ_10_). As was the case for HP dp12 and dp24, this resulted in a larger difference in EED values between the experimentally studied and simulated HP dp68 molecules (percentage error up to 21.1%).

Assuming that HP dp12 is a representative of shorter chains of HP, HP dp68—representative of long HP chains, and HP dp24 of medium-length chains, several observations can be made from the conducted analyses. Compared to medium-length and long HP, HP dp12 exhibits a greater tendency to adopt extended conformations. Unsurprisingly, the high importance of the repulsive electrostatic interactions between the CG sugar residues seems to be the most important energy term for HP dp12, causing the extension of the HP chain. While HP dp24 achieved *R*_g_ and EED close to experimental values for all tested κ_*i*_ values, provided the appropriate modification of the energy term weights, HP dp68 showed a slight preference for higher ion concentrations, corresponding to more compact conformations. The energy term most important to achieve conformations of HP dp68 resemble the experimental ones was the virtual bond stretching energy. Taken together with the importance of the high torsional energy term weight in obtaining conformations of *R*_g_ comparable to experimental data, this suggests a more dynamic and flexible behavior of the long HP chains compared to the shorter HP molecules.

At the same time, it is important to highlight the possible bias toward more coiled conformations of the molecules studied due to the origin of the used parameters. The initial parameters used in the effective energy terms were determined based on all-atom MD simulations of HP using the GLYCAM06 force field and the TIP3P explicit water model.^73^ However, the limitations of different water models in all-atom simulations of GAGs have been demonstrated in some studies: TIP3P may not reproduce the charge distribution with the same quality as more complex solvent models and therefore result in highly bent conformations of HP and other GAGs.^95,96^ This is likely caused by the accumulation of counterions near the GAG molecules, which enabled the relatively close contacts between sugar residues. Therefore, the tendency of HP to adopt coiled and bent conformations when simulated in the CG SUGRES-1P force field may be influenced by the origin of the parameters of the force field used to obtain them.^73^

The length, flexibility, and overall shape of HP chains are important to ensure the formation of correct conformations and allow binding to proteins. The differences between experimental values of both EED and *R*_g_ and those of the CG molecules became more apparent with increasing chain length. The *R*_g_ of the tested HP molecules appeared to be more robust than the EED, i.e., it did not deviate as strongly from the experimental values for molecules simulated using combinations of energy term weights and κ optimized for a better agreement of the EED. Therefore, the parameters for subsequent analyses were chosen among those combinations of energy term weights and κ_*i*_, which resulted in HP conformations characterized by EED close to the experimental chain lengths. As a result, three combinations of κ_*i*_ and energy term weights were chosen, each corresponding to the optimal parameters for HP dp12, dp24, and dp68: κ_2_ and *w*_eel_ = 7, κ_7_ and *w*_eel_ = 7, and finally κ_7_ together with *w*_bond_ = 4.

### 3.2 CG Simulations of Short and Long Free HP Molecules for Selected Weights of Effective Energy Terms and Selected Values of κ

**Figure 5:**
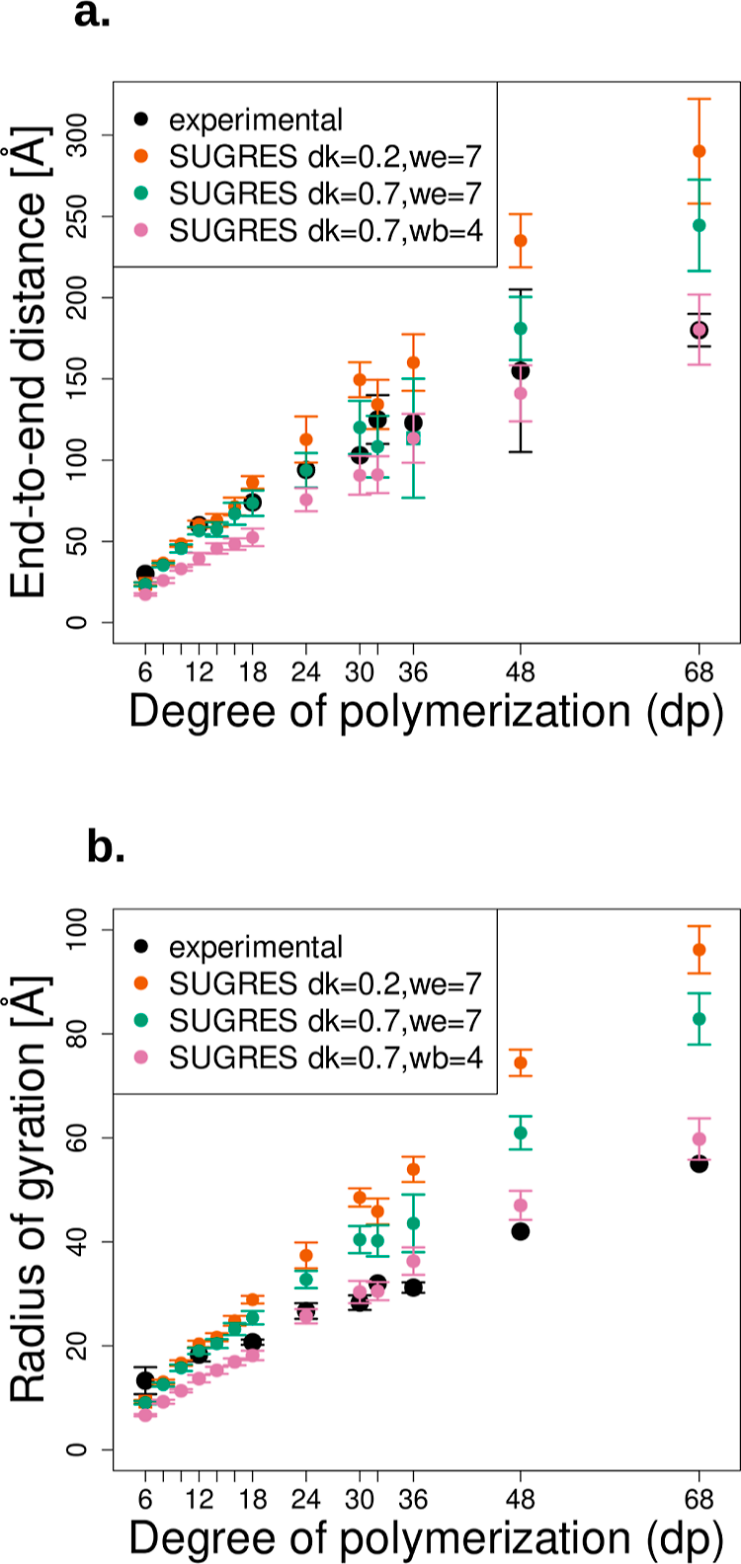
Dependence of the end-to-end distance (EED) (a) and the radius of gyration (*R*_g_) (b) on the effective-energy term weight and ion concentration expressed by the κ parameter for HP dp from 6 to 68. Black points correspond to the experimental values from refs (74) and (75); standard deviation of the experimental values are represented by black bars where available. The EED and *R*_g_ values obtained by the modification of the weights and κ are shown as colored points, with a standard deviation represented by bars of corresponding color.

Simulations of HP of length dp6 to dp68 were conducted in the SUGRES-1P force field using the three combinations of κ_*i*_ and energy term weights mentioned in the previous subsection. The EED of the obtained HP molecules was plotted as a function of the degree of polymerization of HP chains (Figure 5). As could be expected, the parameter set, which previously was optimal for HP dp12, also proved to be better in the simulation of shorter HP chains. The difference in the experimental EED and the EED of CG HP was the smallest for this set of parameters compared to the other parameter sets for HP dp6 and HP dp12 (percentage errors of 20.4 and 0.8% for HP dp6 and dp12, respectively; Table S1). The conformations of medium-long HP molecules (dp18 and dp24) had EED values closest to the experimental ones with parameters optimal for HP dp24 (percentage error of 0.8% for HP dp18 and 0.4% for HP dp24; Table S5). The EED of HP dp30 was estimated with roughly the same percentage error by both the HP dp24-fitted and HP dp68-fitted parameter combinations, with an error of 16.5% for κ_7_ with *w*_eel_ = 7 and an error of 12.0% for κ_7_ and *w*_bond_ = 4. This was also the case for HP dp36, where the percentage error between the experimental and theoretical EED was equal to 7.8% when using either of the aforementioned parameter combinations. Interestingly, the best parameter combination in terms of agreement of experimental and theoretical EED of HP dp32 was the HP dp12-fitted combination of κ_2_ and *w*_eel_ = 7. Both HP dp48 and HP dp68 exhibited a better agreement with the experimentally determined EED when simulated using parameters estimated from tests using HP dp68 (percentage errors of 9.0 and 0.1% for HP dp48 and dp68, respectively).

The *R*_g_ of the short HP dp6 was underestimated in all three tested combinations of κ_*i*_ and weights (Table S6), with a minimum of 30.5% error in the value of *R*_g_ in the case of HP dp12-fitted parameters. In the case of HP dp12, the best parameter combination was κ_7_ with *w*_eel_ = 7, resulting in only 3.9% of error above the experimentally determined value of *R*_g_. For all other HP lengths (HP dp18 to dp68), the best agreement with experimental *R*_g_ was obtained when simulating the molecules using κ_7_ and *w*_bond_ = 4. For HP dp18, dp24, and dp32, the optimal parameter combination underestimated the *R*_g_ of the simulated chains relative to the experimental *R*_g_ (percentage errors of 12.3, 3.8, and 7.2% for dp18, dp24, and dp32, respectively). In simulations of HP dp30, dp36, dp48, and dp68, the *R*_g_ of the obtained conformations lied above the experimentally determined values (percentage errors of 7.2% for HP dp30, 16.3% for HP dp36, 12.0% for HP dp48, and 8.7% for HP dp68). It seems, therefore, that the conformations of HP obtained by simulations using the CG SUGRES-1P force field would be able to reproduce either the degree of extension of the chains, as expressed by the EED, or their radius of gyration, but not both at the same time. In general, the HP dp12-fitted parameters caused the HP molecules to adopt more extended chains, characterized by a higher *R*_g_. The shorter, more coiled chains obtained by conducting the CG simulations with the dp68-fitted parameters were a good choice for the longer chains (from HP dp18 to dp68). The parameters that seemed like a good “middle ground” when considering the agreement with experimental EED resulted in a small difference in the *R*_g_ values of experimental *R*_g_ and the *R*_g_ of the simulated molecules only for HP dp12.

**Figure 6:**
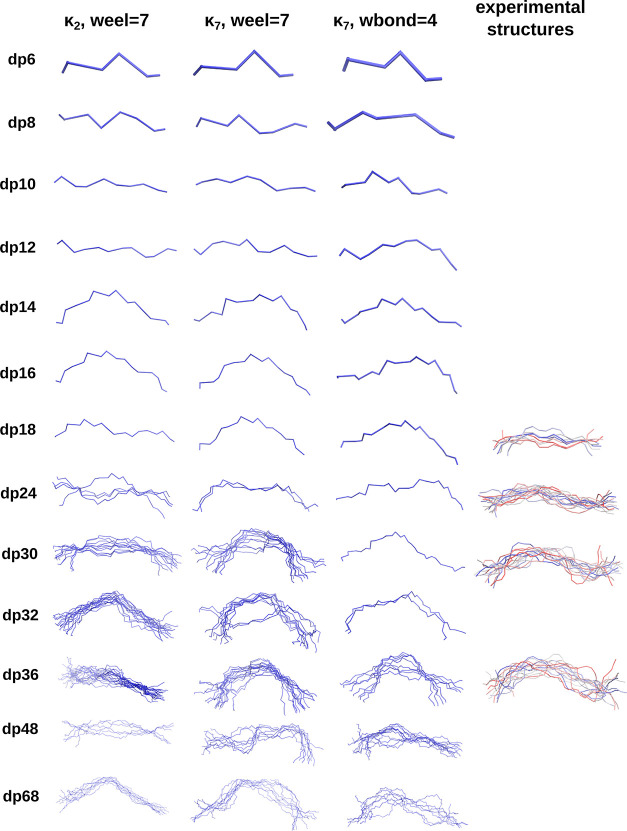
Representative conformations of the CG trajectories of HP conducted using κ_2_ with *w*_eel_ = 7, κ_7_ with *w*_eel_ = 7, and κ_7_ and *w*_bond_ = 4, visualized in trace representations, colored in blue. Additionally, the CG SUGRES-1P representation of the experimentally determined conformations of HP^74^ of HP dp18 (PDB ID: 1IRI), dp24 (PDB ID: 1IRJ), dp30 (PDB ID: 1IRK), and dp36 (PDB ID: 1IRL) in trace representation.

The structures obtained by the CG MD simulations were clustered using the DBSCAN algorithm to identify representative conformations for particular κ_*i*_ and energy term weights, as visualized in Figure 6. The simulated HP molecules adapted coiled conformations independently of the parameter combination used in the CG simulations. In most of the simulations, only one conformation cluster could be identified due to the similarity of the sampled conformations. Importantly, in all three simulation settings and for all of the identified cluster representatives, a kink in the chain appeared for HP longer than dp14, corresponding to the kink observed experimentally, e.g., in ref (74). However, using κ_2_ and *w*_eel_ = 7, fitted for shorter HP chains, the kink became less pronounced for longer HP molecules. At the same time, the overall shape of the other HP molecules did not seem qualitatively too different between the parameters fitted for medium-long and those for long HP chains (κ_7_ and *w*_eel_ = 7). The representative conformations have been compared with the experimentally determined conformations of HP deposited in the RCSB PDB under the IDs: 1IRI, 1IRJ, 1IRK, and 1IRL,^74^ visualized in the SUGRES-1P CG representation, as shown in Figure 6. A striking similarity in the overall shape of the chains of CG HP can be observed—while the simulated chains appear to be slightly more extended than their experimental counterparts, the key feature of the chains, i.e., the kink in the structure, is well preserved in the CG molecules.

Table S7 details the root-mean-square deviation (RMSD) calculated for O4 atoms of the CG HP structures in reference to the first model in the conformations of HP dp18, dp24, dp30, and dp36 in PDB entries 3IRI, 3IRJ, 3IRK, and 3IRL.^74^ The variety of structures obtained from CG simulations lies within the range of the experimentally determined diversity of the dynamic HP molecules. In the case of HP dp18, the RMSD between the ensemble of structures in PDB ID 3IRI(74) reached up to 7.6 Å, which is only 0.3 Å less than the mean RMSD between all of the frames of the CG simulation of HP in reference to the models in 3IRI. This difference in RMSD values increases with the increasing length of the HP chain; however, it remains relatively small. The RMSD obtained during CG simulations in reference to the models in PDB entry 3IRJ is 10.3 Å (Table S7), which is 2.7 Å larger than the RMSD between the experimentally determined conformations in PDB 3IRJ.^74^ In the case of HP dp30 and dp36, the RMSD between the structure models in the respective PDB entries ranged up to 10.1 and 14.5 Å, while the mean RMSD of the HP structures obtained by CG simulations calculated in reference to the models in the respective PDB entries lies at 15.2 and 18.6 Å, respectively. This shows that the CG simulations of the HP chains in the SUGRES-1P force field were able to capture the dynamic behavior of the HP molecules approximately within the ranges of the experimental diversity of HP conformations.^74^ It is important to note that the results of the CG simulation in the SUGRES-1P force field are based purely on theoretical approaches and no constraints based on experimentally determined EED were imposed on the simulated HP chains during the CG simulations. Taking all comparisons of the EED, *R*_g_, and overall shape of the simulated chains into account, κ_7_ together with *w*_eel_ = 7 is likely a good combination of parameters that can be used for the simulation of both short and long HP chains.

## 4 Conclusions

We have extended the SUGRES-1P CG model to the simulation of free HP chains. The effective interaction energy function has been modified compared to ref (73) by extracting the electrostatic interaction energy term from the sum containing all other interaction energies of two sugar residues to enable a direct modification of the corresponding electrostatic energy term weight. This enabled us to obtain more extended HP chain conformations characterized by remarkable similarity to the experimentally determined HP molecules. The estimation of the energy term weights and the κ_*i*_ parameter, describing the ion concentration of the simulated system, enabled us to identify the most important elements of the effective energy function specific to HP chains of different lengths. The results suggest that long HP molecules most likely adopt more coiled conformations, governed predominantly by the electrostatic interaction energy of the charged HP residues. The SUGRES-1P module is fully compatible with the UNICORN model of other biomacromolecule types, consequently providing the potential for simulating and analyzing their interactions with HP. The next step in our work is to thoroughly explore the interaction between HP and particular proteins, providing a significant approach to model large protein systems that contain GAGs and will prove useful in the simulation of the corresponding key biological phenomena, such as collagen reorganization,^97,98^ the maintenance of protein gradients in the presence of GAGs,^99−102^ and GAG-induced amyloidogenesis.^103,104^ The inclusion of parameters for other types of GAGs in the CG SUGRES-1P model will be addressed in future works.

## Supporting Information Available

Debye-Ḧuckel screening factor κ, EED, R_g_, and RMSD (PDF)

## Notes

The SUGRES-1P package is available free of charge at: https://unres.pl/downloads together with example input files.
