# Supplementary material for: Extension of the SUGRES-1P Coarse-Grained Model of Polysaccharides to Heparin
Source: J Chem Theory Comput. 2023 Aug 16;19(17):6023–36. doi: 10.1021/acs.jctc.3c00511 (PMC10500997; doi:10.1021/acs.jctc.3c00511)
Supplement: Supplementary file 1 — ct3c00511_si_001.pdf [file ct3c00511_si_001.pdf]

Supporting Information
Extension of the SUGRES-1P Coarse-Grained Model of Polysaccharides to Heparin

Annemarie Danielsson[1], Sergey A. Samsonov[1], Adam Liwo[1], and Adam K. Sieradzan[1,*]

[1]Faculty of Chemistry, University of Gdansk, ul. Wita Stwosza 63, 80-308 Gdańsk, Poland.
[*]Corresponding author: adam.sieradzan@ug.edu.pl

	GlcNS6S-GlcNS6S	GlcNS6S-IdoA2S	IdoA2S-IdoA2S
κ_0	0.00	0.00	0.00
κ_1	0.10	0.10	0.04
κ_2	0.20	0.20	0.08
κ_3	0.30	0.30	0.12
κ_4	0.40	0.40	0.16
κ_5	0.50	0.50	0.20
κ_6	0.60	0.60	0.25
κ_7	0.70	0.70	0.29
κ_8	0.80	0.80	0.33
κ_9	0.90	0.90	0.37
κ_{10}	0.10	0.10	0.41

Table S1: Values of the Debye-Hückel screening factor κ, specific to each pair of interacting HP residues, optimized during CG simulations in the SUGRES-1P force field. GlcNS6S - 6-*O*-sulfated and *N*-sulfated glucosamine, IdoA2S - 2-*O*-sulfated iduronic acid. All values are approximated to two decimal places.

	Best agreement with experimental EED		Best agreement with experimental R_g	
1	κ_2, w_{eel}=7	EED = 60.1 Å R_g = 20.3 Å	κ_3, w_{eel}=4	EED = 53.2 Å R_g = 18.1 Å
2	κ_3, w_{eel}=9	EED = 60.3 Å R_g = 20.3 Å	κ_2, w_{eel}=5	EED = 54.5 Å R_g = 18.5 Å
3	κ_5, w_{eel}=10	EED = 59.9 Å R_g = 20.1 Å	κ_2, w_{eel}=4	EED = 53.0 Å R_g = 18.1 Å
4	κ_4, w_{eel}=10	EED = 59.6 Å R_g = 20.2 Å	κ_1, w_{eel}=4	EED = 53.1 Å R_g = 18.5 Å
5	κ_0, w_{eel}=4	EED = 60.5 Å R_g = 20.3 Å	κ_0, w_{eel}=2	EED = 52.6 Å R_g = 18.0 Å
6	κ_2, w_{eel}=8	EED = 60.8 Å R_g = 20.5 Å	κ_4, w_{eel}=5	EED = 53.1 Å R_g = 18.0 Å
7	κ_4, w_{eel}=9	EED = 59.2 Å R_g = 19.9 Å	κ_4, w_{eel}=6	EED = 54.9 Å R_g = 18.6 Å
8	κ_0, w_{eel}=3	EED = 59.0 Å R_g = 20.0 Å	κ_3, w_{eel}=5	EED = 55.7 Å R_g = 18.6 Å
9	κ_3, w_{eel}=10	EED = 61.0 Å R_g = 20.6 Å	κ_5, w_{eel}=6	EED = 49.6 Å R_g = 17.9 Å
10	κ_1, w_{eel}=6	EED = 61.1 Å R_g = 20.6 Å	κ_6, w_{eel}=4	EED = 52.6 Å R_g = 17.9 Å
experimental values	EED = 60.0 Å, R_g = 18.3 ± 1.3 Å			

Table S2: Values of EED [Å] and R_g [Å] for HP dp12 together with the κ_i and energy term weight modification used in the CG simulations in the SUGRES-1P force field in which the corresponding EED and R_g values were obtained; where: w_{eel} - electrostatic energy term weight. Additionally, the EED and R_g values in [Å] of the experimentally-investigated HP dp12 in [74]. All values are rounded up to one decimal place.

	Best agreement with experimental EED		Best agreement with experimental R_g	
1	κ_7, w_{eel}=7	EED = 93.7 Å R_g = 32.8 Å	κ_6, w_{bond}=2	EED = 79.8 Å R_g = 26.6 Å
2	κ_5, w_{eel}=5	EED = 94.4 Å R_g = 32.6 Å	κ_0, w_{vdw}=10	EED = 73.5 Å R_g = 26.6 Å
3	κ_4, w_{eel}=7	EED = 93.5 Å R_g = 33.0 Å	κ_2, all weights = 1	EED = 77.9 Å R_g = 26.9 Å
4	κ_9, w_{eel}=4	EED = 93.0 Å R_g = 31.9 Å	κ_7, w_{bond}=2	EED = 80.3 Å R_g = 26.9 Å
5	κ_7, w_{eel}=2	EED = 95.0 Å R_g = 31.3 Å	κ_1, w_{vdw}=4	EED = 78.0 Å R_g = 26.9 Å
6	κ_5, w_{eel}=7	EED = 95.1 Å R_g = 32.6 Å	κ_4, w_{bond}=3	EED = 81.1 Å R_g = 26.5 Å
7	κ_9, w_{eel}=3	EED = 92.9 Å R_g = 31.5 Å	κ_2, w_{bond}=3	EED = 81.2 Å R_g = 26.4 Å
8	κ_{10}, w_{eel}=8	EED = 95.2 Å R_g = 33.7 Å	κ_1, w_{bond}=3	EED = 82.8 Å R_g = 27.0 Å
9	κ_0, w_{eel}=2	EED = 92.7 Å R_g = 30.5 Å	κ_5, w_{bond}=3	EED = 81.8 Å R_g = 26.4 Å
10	κ_6, w_{eel}=4	EED = 92.6 Å R_g = 31.7 Å	κ_0, w_{bond}=4	EED = 84.8 Å R_g = 27.1 Å
experimental values	EED = 94.0 Å, R_g = 26.7 ± 1.5 Å			

Table S3: Values of EED [Å] and R_g [Å] for HP dp24 together with the κ_i and energy term weight modification used in the CG simulations in the SUGRES-1P force field in which the corresponding EED and R_g values were obtained; where: w_{eel} - electrostatic energy term weight, w_{bond} - virtual bond-stretching energy term, w_{vdw} - the weight of the the interaction energy of sugar residue pairs (excluding the Coulombic electrostatic energy term). Additionally, the EED and R_g values in [Å] of the experimentally-investigated HP dp24 in [74]. All values are rounded up to one decimal place.

	Best agreement with experimental EED		Best agreement with experimental R_g	
1	κ_7, w_{bond}=4	EED = 180.2 Å<br>R_g = 59.8 Å	κ_0, w_{tor}=8	EED = 142.0 Å<br>R_g = 54.7 Å
2	κ_2, w_{bond}=4	EED = 179.6 Å<br>R_g = 60.2 Å	κ_4, w_{bond}=10	EED = 146.3 Å<br>R_g = 55.4 Å
3	κ_5, w_{bond}=2	EED = 179.2 Å<br>R_g = 61.5 Å	κ_3, w_{bond}=6	EED = 158.8 Å<br>R_g = 55.4 Å
4	κ_{10}, w_{bond}=5	EED = 180.7 Å<br>R_g = 62.6 Å	κ_2, w_{vdw}=4	EED = 173.2 Å<br>R_g = 55.5 Å
5	κ_1, wvdw=7	EED = 179.2 Å<br>R_g = 60.0 Å	κ_4, w_{bond}=7	EED = 149.3 Å<br>R_g = 54.3 Å
6	κ_4, w_{bond}=2	EED = 181.1 Å<br>R_g = 63.8 Å	κ_7, w_{bond}=6	EED = 151.1 Å<br>R_g = 55.8 Å
7	κ_8, w_{bond}=7	EED = 181.3 Å<br>R_g = 57.9 Å	κ_5, w_{bond}=7	EED = 152.9 Å<br>R_g = 55.8 Å
8	κ_{10}, w_{bond}=8	EED = 178.7 Å<br>R_g = 59.0 Å	κ_{10}, w_{bond}=9	EED = 160.3 Å<br>R_g = 54.2 Å
9	κ_6, w_{bond}=4	EED = 181.4 Å<br>R_g = 59.5 Å	κ_5, w_{bond}=8	EED = 161.1 Å<br>R_g = 54.1 Å
10	κ_8, w_{bond}=5	EED = 181.5 Å<br>R_g = 60.6 Å	κ_8, w_{bond}=8	EED = 164.1 Å<br>R_g = 55.9 Å
experimental values	EED = 180.0 ± 10.0 Å, R_g = 55.0 Å			

Table S4: Values of EED [Å] and R_g [Å] for HP dp68 together with the κ_i and energy term weight modification used in the CG simulations in the SUGRES-1P force field in which the corresponding EED and R_g values were obtained; where: w_{bond} - virtual bond-stretching energy term, w_{tor} - virtual bond-torsional angle deformation energy, w_{vdw} - the weight of the interaction energy of sugar residue pairs (excluding the Coulombic electrostatic energy term). Additionally, the EED and R_g values in [Å] of the experimentally-investigated HP dp68 in [75]. All values are rounded up to one decimal place.

HP length (dp)	EED [Å] at κ_2, w_{eel}=7	EED [Å] at κ_7, w_{eel}=7	EED [Å] at κ_7, w_{bond}=7	experimental EED [Å] [1, 2]
dp6	23.9 ± 1.0	23.5 ± 1.1	17.3 ± 0.8	30.0
dp8	36.6 ± 1.4	35.4 ± 1.2	25.9 ± 1.6	—
dp10	48.5 ± 1.9	45.6 ± 2.5	33.1 ± 1.1	—
dp12	60.5 ± 2.3	56.6 ± 2.3	39.3 ± 3.6	60.0
dp14	62.9 ± 4.0	57.4 ± 4.4	45.6 ± 3.2	—
dp16	71.5 ± 5.5	67.0 ± 6.7	48.3 ± 3.5	—
dp18	86.3 ± 3.9	73.5 ± 7.8	52.5 ± 5.4	74.0
dp24	112.7 ± 14.2	93.7 ± 10.6	75.6 ± 7.1	94.0
dp30	149.5 ± 10.7	120.1 ± 16.3	90.6 ± 11.9	103.0
dp32	134.3 ± 15.2	108.3 ± 18.9	91.0 ± 11.3	125.0 ± 15.0
dp36	160.1 ± 17.4	113.5 ± 36.6	113.4 ± 15.1	123.0
dp48	235.0 ± 16.4	181.0 ± 19.4	141.1 ± 17.2	155.0 ± 50.0
dp68	290.1 ± 32.2	244.4 ± 28.1	180.3 ± 21.6	180.0 ± 10.0

Table S5: Values of the EED and standard deviation of the HP molecules simulated in the CG SUGRES force field at different salinity levels expressed by the κ parameter and with modifications of the energy term weights (w_{eel}: electrostatic energy term weight, w_{bond}: virtual bond-stretching energy term weight), and of the experimentally-determined EED values by [74, 75]. All values are rounded up to one decimal place.

HP length (dp)	R_g [Å] at κ_2, w_{eel}=7	EED [Å] at κ_7, w_{eel}=7	R_g [Å] at κ_7, w_{bond}=7	experimental R_g [Å] [1, 2]
dp6	9.3 ± 0.3	9.1 ± 0.3	6.7 ± 0.2	13.3 ± 2.6
dp8	13.1 ± 0.4	12.5 ± 0.4	9.2 ± 0.4	—
dp10	16.7 ± 0.5	15.8 ± 0.6	11.3 ± 0.3	—
dp12	20.4 ± 0.6	19.0 ± 0.6	13.7 ± 0.7	18.3 ± 1.3
dp14	21.7 ± 0.8	20.4 ± 0.8	15.3 ± 0.7	—
dp16	24.8 ± 0.9	23.2 ± 1.2	16.9 ± 0.7	—
dp18	28.9 ± 0.7	25.4 ± 1.3	18.1 ± 0.9	20.7 ± 0.5
dp24	37.4 ± 2.5	32.8 ± 1.6	25.7 ± 1.4	26.7 ± 1.5
dp30	48.5 ± 1.8	40.4 ± 2.6	30.3 ± 2.1	28.3 ± 1.4
dp32	45.9 ± 2.5	40.2 ± 3.0	30.5 ± 1.7	32.0
dp36	54.0 ± 2.4	43.6 ± 5.5	36.3 ± 2.6	31.2 ± 1.0
dp48	74.4 ± 2.5	61.0 ± 3.2	47.0 ± 2.8	42.0
dp68	96.2 ± 4.5	82.9 ± 4.9	59.8 ± 4.0	55.0

Table S6: Values of the R_g and standard deviation of the HP molecules simulated in the CG SUGRES force field at different salinity levels expressed by the κ parameter and with modifications of the energy term weights (w_{eel}: electrostatic energy term weight, w_{bond}: virtual bond-stretching energy term weight), and of the experimentally-determined R_g values by [74, 75]. All values are rounded up to one decimal place.

	mean RMSD to first model in the PDB [Å]	mean RMSD to all models in the PDB [Å]
HP dp18	8.7 ± 1.3	7.9 ± 0.8
HP dp24	9.1 ± 0.8	10.3 ± 1.0
HP dp30	16.4 ± 1.1	15.2 ± 1.2
HP dp36	19.2 ± 2.0	18.6 ± 2.3

Table S7: Root mean square deviation (RMSD) [Å] of the entire coarse-grained HP trajectory in reference to the experimentally-determined 3D structure of HP for dp18, dp24, dp30 and dp36 (PDB IDs: 3IRI, 3IRJ, 3IRK, 3IRL) [74]. The RMSD was calculated for $O4$ atoms of the glycosidic linkages, which serve as anchor points for the coarse-grained HP polysaccharide chains.
